# Influence of Waste Engine Oil Addition on the Properties of Zeolite-Foamed Asphalt

**DOI:** 10.3390/ma12142265

**Published:** 2019-07-15

**Authors:** Agnieszka Woszuk, Michał Wróbel, Wojciech Franus

**Affiliations:** Department of Geotechnics, Faculty of Civil Engineering and Architecture, Lublin University of Technology, Nadbystrzycka 40, 20-618 Lublin, Poland

**Keywords:** asphalt, zeolite, dynamic viscosity, waste engine oil, penetration, softening point

## Abstract

The previous studies on asphalt mix and asphalt with waste engine oil addition indicate the possibility of using this type of waste material for the construction of road pavements. The research presented in this paper aimed at the preliminary assessment of possible waste engine oil (WEO) addition to the asphalts foamed with water-soaked zeolites. In this research, synthetic zeolite Na-P1 and natural clinoptilolite were used. In order to improve the foaming effect, the zeolites were soaked with water before dispensing to the asphalt, in the amount of 75% asphalt weight for Na-P1 and 25% for clinoptilolite. The tests were performed for one type of waste engine oil—5W40 and two type of binders: 20/30 and polymer modified 25/55-60. The asphalt parameters such as the dynamic viscosity, penetration and softening point were determined with the addition of WEO and zeolites in the concentration of 0%, 3%, 5%, 7% for both materials. It was found that the WEO addition lowers the viscosity and softening point of asphalt but increases penetration. The zeolite addition affected the change of these parameters to a minor extent or was statistically irrelevant. The chemical analysis of the asphalt samples with WEO addition performed with the X-ray Fluorescence method did not show a significant amount of heavy metals which would increase the probability of low-temperature cracking. The analysis of the results indicates the possibility of using zeolite-foamed asphalt technology with WEO addition.

## 1. Introduction

The construction of road surfaces is associated with a significant consumption of natural resources, including mineral aggregates and crude oil, as well as the emission of hazardous compounds into the atmosphere during the process of asphalt mix production. Through the use of waste materials and innovative technologies, there is a chance to make road construction not only environmentally friendly but also less cost-intensive, while ensuring the appropriate durability of the pavement as well as comfort and safety for users [1,2]. Long-term research results indicate that various waste materials can be used in the asphalt mix instead of the natural aggregate, such as: Construction and demolition waste (CDW) [3,4,5], ceramic aggregates [6,7,8] and fly ash [9,10]. 

In recent years, accelerated degradation of road pavements has been a major problem occurring in Poland [11]. It is related to an increased level of heavy duty traffic and a change of the road traffic structure [12]. The increasing amount of reclaimed asphalt pavement (RAP) is the consequence of these changes. According to the rules of sustainable development, the best way to manage RAP is to re-use it in asphalt pavements, especially in the field of the newly produced asphalt mix. The changed rheological properties of asphalt under the influence of oxidation constitute a significant issue in using RAP for these mixes. The increase of stiffness may have a major negative effect on the asphalt pavement durability [13,14]. Rejuvenating agents, which restore the chemical structure of aged asphalt by providing lost aromatic constituents and reducing the overall viscosity of the binder, are used for improving the properties of mix asphalt with RAP [15,16]. One of the potential municipal waste materials that can be used to rejuvenate RAP is waste engine oil (WEO) from cars [17,18,19]. The structure of WEO resembles the molecular structures of asphalt with sufficient aromatic content, which leads to coherent bonding by altering the constituents and rejuvenating the aged asphalt [20,21]. Bio-oil has a similar structure and it can be also successfully used as a rejuvenating agent in mix asphalt [22,23,24]. According to the latest research WEO allows not only to increase the amount of RAP addition in newly designed mixes [25], but also adjusts their properties such as: Fatigue resistance [26], workability [27], indirect tensile strength and moisture susceptibility [17] as well as reduces ductile performance [28]. Waste engine oil may also partially replace virgin asphalt binder. The WEO addition causes softening effect, improves elasticity and recovers properties of base asphalt [29].

Another method that allows increasing the percentage of RAP in new mixes is the warm mix asphalt technology (WMA), which is produced at a temperature lowered by 20 °C–40 °C [30,31]. Lower production temperature positively affects the slowdown of asphalt binder aging, which counteracts the stiffening of layers containing waste materials. Slowed aging of the asphalt compensates the age of the recycled aggregate binder, similar to using softer asphalt. Owing to better workability, warm-mix asphalts may contain higher RAP addition [32]. The research on WMA was performed with the addition of up to 100% RAP. The results indicate good workability in temperatures as low as 110 °C [33].

The aim of this work is to assess the possibility of using waste engine oil in foamed asphalt technology with zeolite addition based on dynamic viscosity, penetration and softening point tests results. According to the fact that WEO is used as a softening additive of asphalt contained in reclaimed asphalt pavement, the conducted research also shows whether there is a potential possibility of using WMA technology with foamed bitumen for asphalt mix with RAP production.

## 2. Experimental Materials

### 2.1. Asphalts

Two types of bitumen were used in the tests: 20/30 asphalt penetration grade and bitumen modified with polymers PMB 25/55-60.

The basic properties of asphalt binders and their fraction composition are presented in Table 1.

The 20/30 asphalt is a hard road bitumen. According to its high softening point and high susceptibility to low temperature cracking, it is recommended for use only in bonding layers and high stiffness modulus base layers under favourable climate regions. Under the Polish climate conditions this type of binder is used occasionally. Considering common petroleum asphalts, the 35/50 penetration grade asphalt is usually used for road pavements. In the research, hard road bitumen 20/30 was chosen, as its properties correspond to asphalt 35/50 after over a dozen or so years of usage in pavement. 

The PMB 25/55-60 asphalt is a popular road bitumen modified with polymers in Poland. It is used for asphalt base layers and high stiffness modulus asphalt concrete. It may also be used in stone mastic asphalt (SMA) layers on the road sections loaded with slow and heavy traffic. New polymer-modified binders are frequently used for improving the properties of mix asphalt with RAP. For that reason the WEO addition impact on foaming effect of this type of binder was additionally verified in the research.

### 2.2. Waste Engine Oil

Waste engine oil used in this study corresponds to type 5W40. The research material was acquired from a local car service.

### 2.3. Zeolites

**Figure 1:**
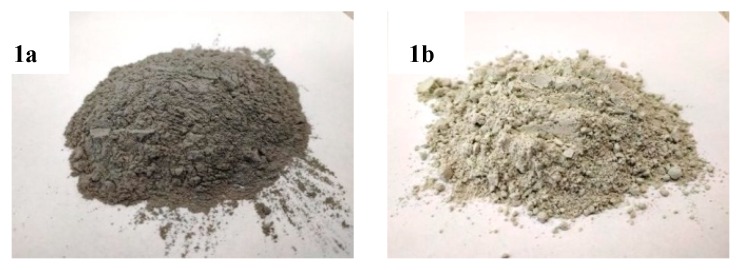
Zeolite materials used in the research: Na-P1 (**a**), clinoptilolite (**b**).

The zeolites used in this study are presented in Figure 1.

**Figure 2:**
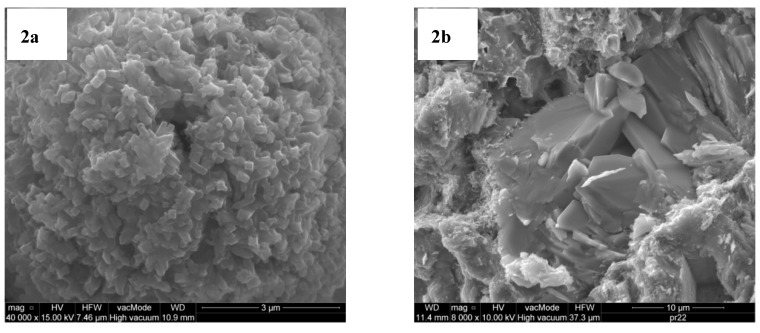
Scanning electron microscopy (SEM) images of Na-P1 (**a**) and clinoptilolite (**b**).

These materials represent two different structure topologies. Na-P1 is a synthetic zeolite obtained in the hydrothermal reaction converting the F-class fly ash and aqueous solution of sodium hydroxide. The pureness of the obtained product was about 70%. Na-P1 is a gismonde-like structure (GIS), which is built out of two four-membered rings forming an eight-membered channel of 3.1 × 4.5 Å and 2.8 × 4.8 Å. NaP1 zeolite forms fine plate-habit grains. Their maximum dimensions are 1 µm of length, 0.2 µm of width and 0.1 µm of thickness. Crystals of the Na-P1 zeolite create rosette-structure clusters (Figure 2a).

The second zeolite – natural clinoptilolite (ZN-C) was acquired from a Ukrainian deposit in the form of zeolitic tuff, which was composed of 75% pure clinoptilolite, cristobalite, quartz, feldzspar and clay minerals such as montmorillonite and illite. ZN-C has a heulandite-like structure (HEU) which is built of two-dimensional channels formed by eight-membered rings of 4.1 × 4.1 Å and 10-membered rings of 2.8 × 4.8 Å. Clinoptilolite also forms plate-habit grains, sometimes hexagonal, however the dimensions are significantly greater than the Na-P1 zeolite (10 × 15 × 0.2 µm) (Figure 2b). Apart from the difference in the mineral structure of the zeolites, they also differ in particle size distribution and the textural parameters [34].

Bound water called zeolite water constitutes an important structural element of zeolites. The way of its release from the crystal structure has a direct impact on the asphalt foaming effect. The character and intensity of the zeolite water release observed in the thermal curves is significantly different for these materials. The endothermic effect is observed up to the temperature of 250 °C, which is of particular interest for asphalt foaming. The maximum effect for the Na-P1 zeolite occurs at the temperature of 120 °C whereas for the natural zeolite—at 190 °C. Both discussed effects are accompanied by the distinct mass reduction (TG curve). In the case of the Na-P1 zeolite mass reduction, it amounts to 20.4%, whereas in the case of clinoptilolite—only to 7.7% [35]. The amount of “zeolite water” released in the temperature range of the asphalt mix production may be insufficient to induce the foaming effect of asphalt binders. In order to improve the effective foaming of bitumens, zeolite materials were additionally soaked with water.

**Figure 3:**
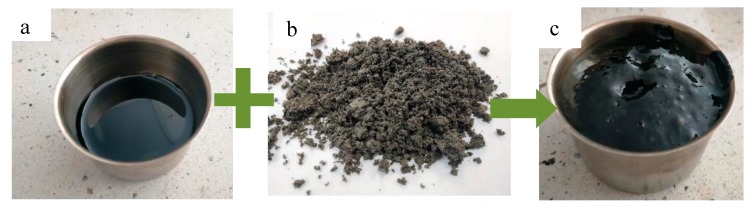
(**a**) 20 grams of 20/30 asphalt in a temperature of 135 °C; (**b**) Na-P1 zeolite soaked with water; (**c**) foaming effect of asphalt caused by water releasing from the zeolite.

The foaming effect of asphalt binder with the addition of water-soaked zeolite is presented in Figure 3. 

## 3. Research Method

### 3.1. Dynamic Viscosity 

The dynamic viscosity tests were performed using a Brookfield’s viscometer according to ASTM D 4402, at a temperature of 135 °C, in reference to paving and compacting the asphalt mix. The test consists of calculating the ratio of shear stress caused by the rotating spindle to its rotational speed.

The measurements were performed for pure asphalt and for the asphalt with additives of the WEO and zeolites soaked in water. The water saturation of zeolites in relation to the dry mass was 75% and 25% w/m for Na-P1 and clinoptilolite, respectively. Both materials were added to the bitumens in the amount of 3%, 5% and 7% in relation to the mass. After heating the asphalt in the oven to the testing temperature, oil was added to the specimen and mixed manually until the mixture became homogeneous. Afterwards, the water-soaked zeolite was added and mixed manually again. Next, the samples were transferred to the viscometer and stabilised for 15 min. The viscosity measurements were taken at the following time intervals: 15, 30, 45 min, counted from the moment of specimen placement in the viscometer. The tests were conducted three times for every combination of asphalt-zeolite-WEO in order to evaluate the repeatability of the obtained results. Each test was performed on a separate sample. The final result is the average of three partial measurements.

### 3.2. Softening Point

The softening point tests were performed according to PN-EN 1427:2009. The softening point is the asphalt temperature at which the specimen located in a standardized ring and heated under certain conditions reaches the base of the device (overcome vertical distance of 25.0 mm ± 0.4 mm) under the weight of steel ball. The test was performed in water with the initial temperature of 5 °C, which rose steadily at a rate of 5 °C/min. According to the standard records, the final result is an average of two measurements rounded to 0.2 °C. 

### 3.3. Penetration 

The penetration tests were performed in accordance to PN-EN 1426:2009. Asphalt penetration is the depth at which the penetrating needle is immersed in the asphalt specimen under the load of 100 g within 5 s at a determined temperature of 25 °C. The final result is an average of three measurements performed on each specimen. 

### 3.4. XRF

The X-ray fluorescence spectrometry (XRF) method was used in order to determine the chemical composition of the asphalt with WEO addition. An EDXRF spectrometer was used, based on the principle of energy dispersion. An EDXRF system typically consists of three main components: An excitation source, a spectrometer/detector as well as data collection and processing unit. In the process, a radiation source illuminates the sample, a detector records the radiation received from the tested sample and thanks to the principle of dispersion or separation reads the values of radiation energy characteristic for particular elements. After the test, the qualitative and quantitative analyses are performed in which elements are identified and the intensity of their graph peaks is determined. In this type of analysis, the intensity of the recorded line corresponds with the area under the peak. The WEO sample and both the 20/30 and 25/55-60 asphalts with 0%, 3%, 5%, 7% WEO addition were tested with this method.

## 4. Results and Discussion

### 4.1. Dynamic Viscosity of Asphalt

**Figure 4:**
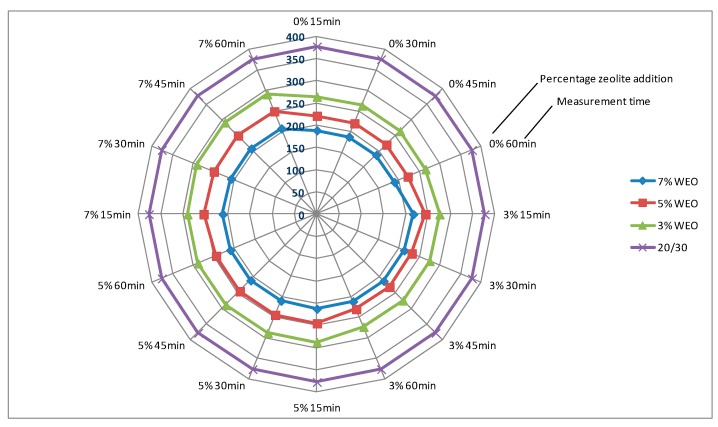
The results of dynamic viscosity tests for the 20/30 asphalt with the addition of WEO and the Na-P1 zeolite measured at 160 °C (mPa·s).

**Figure 5:**
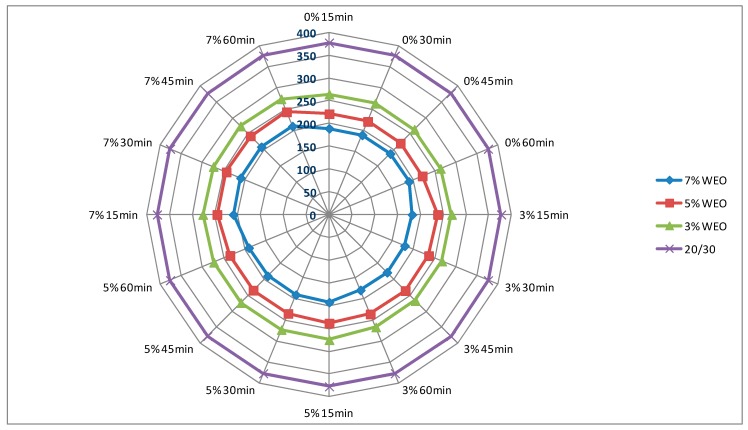
The results of dynamic viscosity tests for the 20/30 asphalt with the addition of WEO and clinoptilolite zeolite measured at 160 °C (mPa·s).

**Figure 6:**
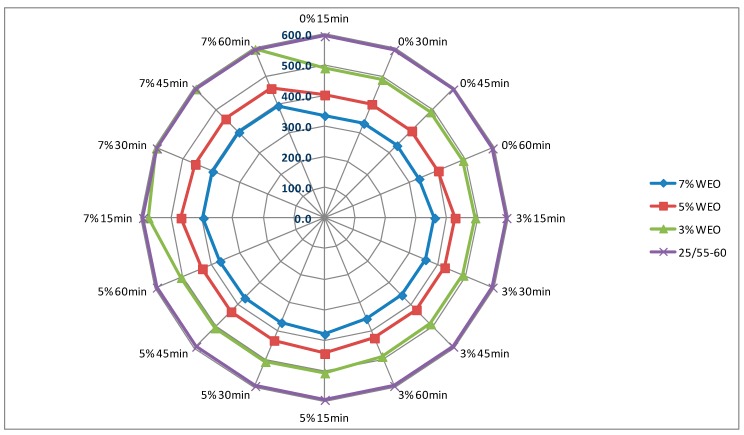
The results of dynamic viscosity tests for the 25/55-60 asphalt with the addition of WEO and the Na-P1 zeolite measured at 160 °C (mPa·s).

**Figure 7:**
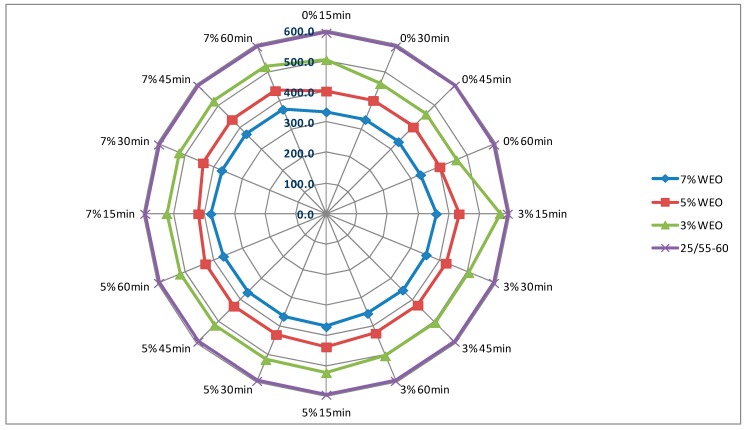
The results of dynamic viscosity tests for the 25/55-60 asphalt with the addition of WEO and clinoptilolite zeolite measured at 160 °C (mPa·s).

The results of dynamic viscosity tests for bitumens with the addition of the WEO and zeolites are presented in Figure 4, Figure 5, Figure 6 and Figure 7. The lines in Figure 4, Figure 5, Figure 6 and Figure 7 in the color green, red and blue determines the viscosity level of asphalt with the addition of WEO (according to the legend). The purple line determines the viscosity of the reference asphalt without additives. The % mark on the external outline of the charts (black font) is the percentage addition of the zeolite material in relation to the weight of the asphalt. The minute designation (black font) refers to the moment of viscosity measurement counted from the moment of specimen placement in the viscometer (Measurement time and percentage zeolite addition has been marked additionally on the first figure with the viscosity results—Figure 4). The viscosity value in the mPa·s unit is presented with a dark blue font.

The viscosity of the 20/30 asphalt was 377 mPa·s. After adding 3% of WEO, viscosity decreased to 264 mPa·s, 5%—to 221 mPa·s, while in the case of 7%—to 189 mPa·s (Figure 4 and Figure 5). The viscosity of the polymer-modified 25/55-60 asphalt was 593.5 mPa·s. With the WEO addition, the obtained viscosity results reached: 3% WEO—461 mPa·s, 5% WEO—402 mPa·s and 7% WEO—334 mPa·s (Figure 6 and Figure 7). The highest percentage change of viscosity was observed with 3% added WEO, for the 20/30 bitumen, it decreased 30% in relation to the initial value and 22% for 25/55-60. Viscosity decreased as the WEO addition increased; however, the dynamics of the changes was lower and the results were from 15% to 16% for the 20/30 asphalt and from 13% to 17% for the 25/55-60 asphalt, respectively. According to the FTIR analysis, Liu et al. reported that the asphalt with the WEO viscosity decrease is the effect of reduced amounts of LMS and carbonyl functional groups [36]. Shoukat and Yoo pointed out that decreasing the binder viscosity by introducing additional oil fraction in asphalt colloidal systems, could result in an improved resistance to thermal cracking [18].

In the bitumen samples with WEO foamed by zeolites, the viscosity generally increased along with the zeolite addition (Figure 4, Figure 5, Figure 6 and Figure 7). The phenomenon should be considered natural, as zeolites have the form of an insoluble solid, conversely to organic or chemical additives, which are often completely soluble and form a homogenous liquid with asphalt [37]. Similar correlation was obtained in the research on the asphalts foamed with zeolites and mesoporous materials soaked with water [35,38]. A characteristic trend in the dynamics of changes cannot be determined along with the increase of the zeolite addition. Considering the type of zeolite additive, the samples foamed with 3% of synthetic zeolite Na-P1 had the viscosity changed by 3.6% to 12.5%. The dynamics of changes with the rise of the zeolite addition was from −1.0% to 17.4% (Figure 4 and Figure 6). Using 3% of the natural zeolite in relation to the weight of the asphalt with the WEO addition, the change of viscosity between −5.2% and 9.4% was observed. Increasing the amount of this foaming additive led to further changes of viscosity from −6.2% to 9.3% (Figure 5 and Figure 7). Comparing the viscosity results of the WEO modified asphalt, the samples with the 7% zeolite addition had higher viscosity than before the foaming process.

Similarly as in the bitumens foamed by the addition of zeolites and mesoporous materials, a decrease in viscosity over time was obtained for each combination of asphalt-zeolite-WEO [36,38]. The highest level of changes occurred after 30 min of the test (45 min after mixing asphalt with zeolite). After 45 min, the viscosity level stabilized. The reduction in asphalt viscosity over time was caused by a slow release of the zeolite water.

### 4.2. Softening Point

**Figure 8:**
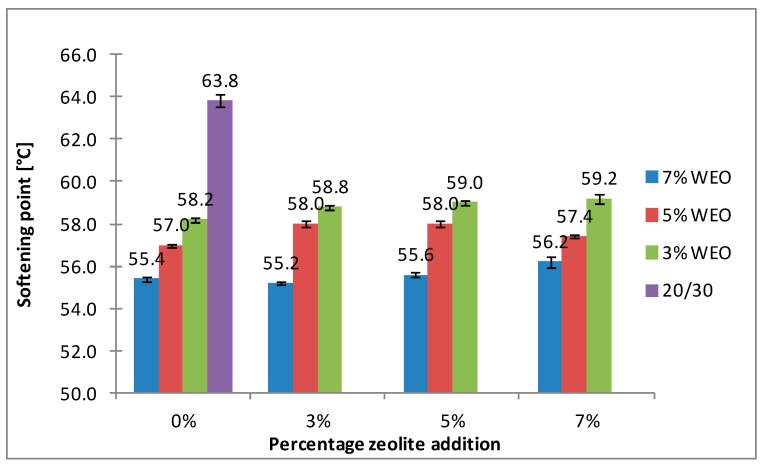
The results of softening point tests for the 20/30 asphalt with the addition of WEO and Na-P1 zeolite.

**Figure 9:**
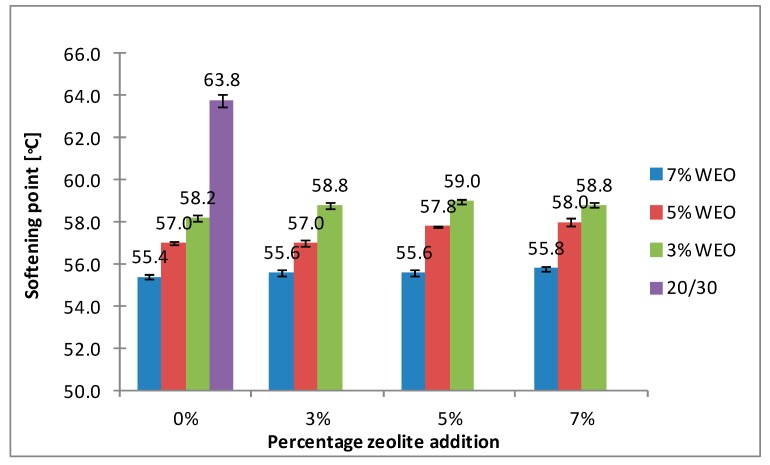
The results of softening point tests for the 20/30 asphalt with the addition of WEO and clinoptilolite zeolite.

**Figure 10:**
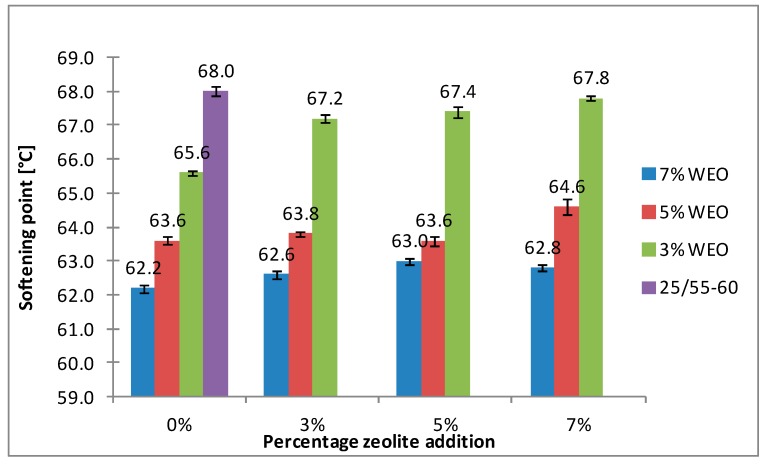
The results of softening point tests for the 25/55-60 asphalt with the addition of WEO and Na-P1 zeolite.

**Figure 11:**
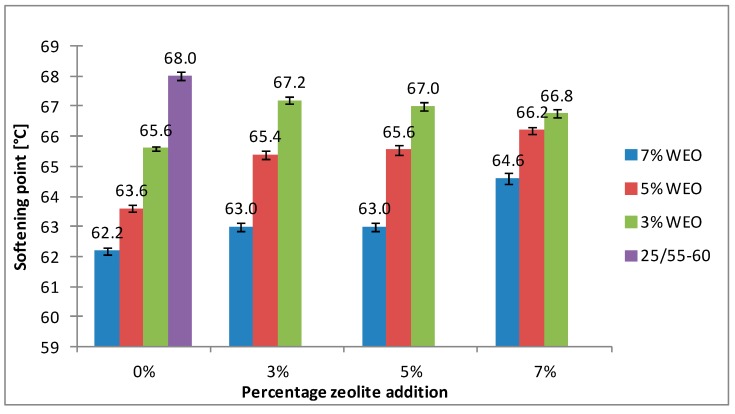
The results of softening point tests for the 25/55-60 asphalt with the addition of WEO and clinoptilolite zeolite.

The results of the softening point for bitumens with the addition of WEO and zeolites are presented in Figure 8, Figure 9, Figure 10 and Figure 11.

The softening point of the 20/30 asphalt was 63.8 °C and 68.0 °C for the 25/55-60 asphalt. Modifying the asphalt binders with 3% WEO addition caused a decrease in the softening point by 5.6 °C for the 20/30 asphalt and by 2.4 °C for PMB. Increasing the percentage of WEO caused a further decrease of the parameter. Similar correlations for road bitumens were obtained by Liu et al. and Zargar et al. [36,39]. However, the dynamics of changes differed from the literature data. In the 20/30 bitumen samples, the WEO addition growth from 3% to 7% caused a decrease in the softening point by 2.8 °C and 3.4 °C for PMB 25/55-60. The softening point change of the asphalt modified with SBS copolymer was lower in comparison to the results acquired by Zargar et al. [39]. Asphalt foaming with water-soaked zeolites did not significantly affect the softening parameter of the 20/30 asphalt (changes from 0.2 °C to 1 °C). In the case of the polymer-modified bitumen, the softening point growth ranged from 1.4 °C to 4 °C in relation to the results of only WEO addition. An increase in the parameter below 1 °C was obtained only in two samples.

The asphalt placed in the road pavement becomes less susceptible to high temperatures under the influence of the oxidation process. In turn, the mixture of waste engine oil can rejuvenate the aged bitumen by decreasing the softening point. Qurashi et al. [19] also found that WEO was good for the softening asphalt and gives it greater flexibility. The negative consequence of such changes is the lower resistance of asphalt pavement to rutting (permanent deformations). It is particularly important in the countries with warm and temperate climate. 

### 4.3. Penetration

**Figure 12:**
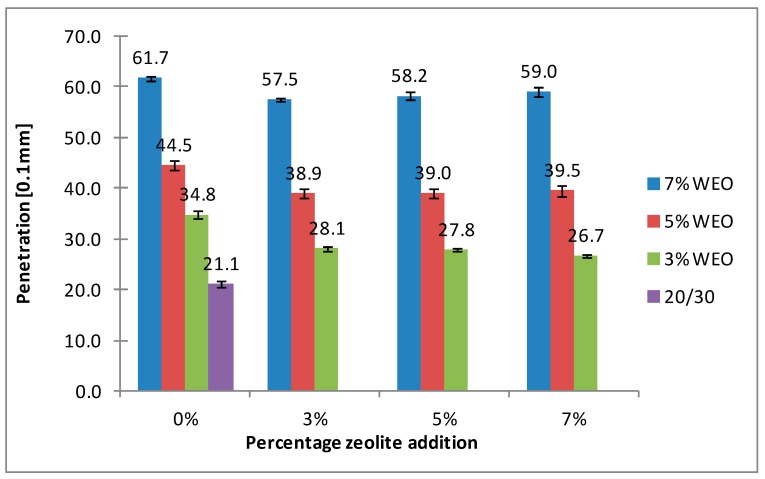
The results of penetration tests for the 20/30 asphalt with the addition of WEO and Na-P1 zeolite measured at 25 °C.

**Figure 13:**
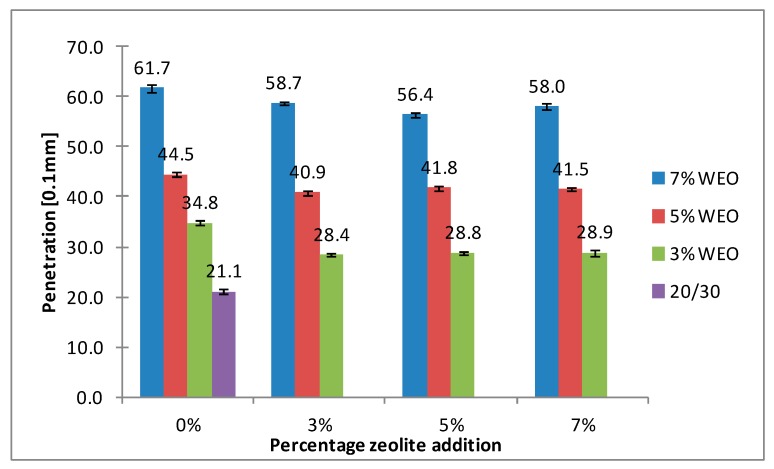
The results of penetration tests for the 20/30 asphalt with the addition of WEO and clinoptilolite zeolite measured at 25 °C.

**Figure 14:**
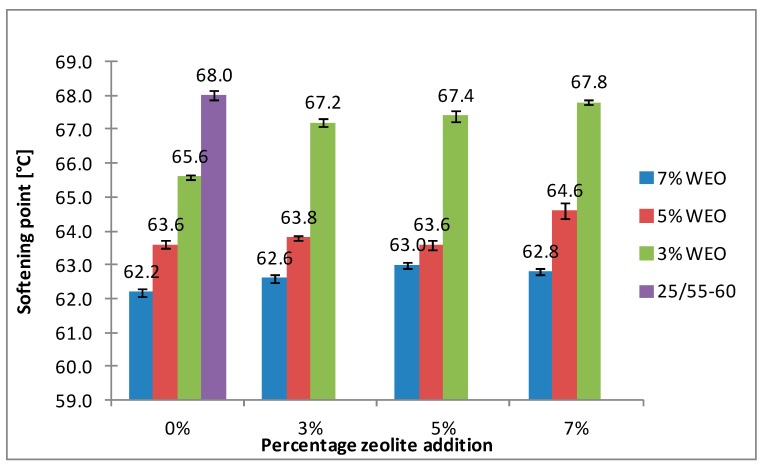
The results of penetration tests for the 25/55-60 asphalt with the addition of WEO and Na-P1 zeolite measured at 25 °C.

**Figure 15:**
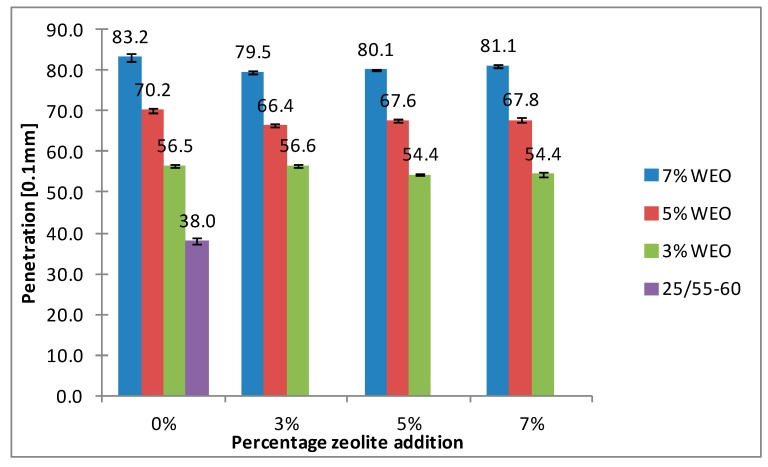
The results of penetration tests for the 25/55-60 asphalt with the addition of WEO and clinoptilolite zeolite measured at 25 °C.

The results of penetration for bitumens with the addition of WEO and zeolites are presented in Figure 12, Figure 13, Figure 14 and Figure 15.

The results presented in Figure 12, Figure 13, Figure 14 and Figure 15 indicate the increase pertaining to the penetration of asphalt binders with the WEO addition. The dynamics of the penetration changes results from the increased amount of WEO additive and has a nearly linear character. In the 20/30 asphalt samples, over a two-fold increase in the tested parameter was obtained using 5% WEO (from 21.2 × 0.1 mm to 44.5 × 0.1 mm). However, in the 25/55-60 asphalt, such level of increase was obtained with 7% WEO addition (from 38 × 0.1 mm to 83.2 × 0.1 mm). The asphalt binders foamed with zeolites had slightly lower penetration. This is caused by the effect of bitumen stiffening after adding a solid in the form of dust. According to Nciri et al., the stiffening process is caused by three mechanisms [40]:

(1) Volumetric-filling reinforcement: The stiffening caused by the presence of rigid inclusions in a less rigid matrix,
(2) Physicochemical reinforcement: The stiffening caused by the interfacial effects between filler particles and asphalt, including adsorption, absorption, and selective sorption.
(3) Particle-interaction reinforcement: The stiffening beyond physicochemical reinforcement and volume filling.

The research conducted by Nciri et al. indicates that the chemical composition of the binder changed after adding ground waste oyster shells. The fraction of aromatic components decreased and the fraction of resins increased. However, the asphaltenes and saturated fractions remained equivalent. The results show that the aromatics in lighter fractions of asphalt were changed to resins. It is possible that zeolite materials interact with the asphalt binder in a similar way, which needs to be confirmed in laboratory tests. 

### 4.4. XRF

The X-ray fluorescence (XRF) technique is the non-destructive method for determining the chemical composition of test materials. In the road construction, it is used to determine the composition of elemental aggregates [41], asphalt [35] additives and modifiers [42]. This method can also be applied to identify the contamination of bitumen by additives characterized by a high amount of heavy metals such as: Zinc (Zn), copper (Cu), molybdenum (Mo) and other elements: Phosphorous (P) and calcium (Ca). The research shows that these types of additives (e.g., Recycled Engine Oil Bottom—REOB) may negatively affect the asphalt quality. The paraffinic nature of the REOB can reduce the adhesion to the aggregate and as a consequence, decrease the resistance of the asphalt mix to water and frost. Metals such as iron, copper, and chromium can act as catalysts in the oxidation of the asphalt cement. Paraffin can precipitate asphaltene, which accelerates the hardening of the asphalt. Such binders are characterized by lower plasticity and higher stress at low temperatures, which results in early and increased thermal cracking [43,44]. An analysis of the chemical composition of asphalt using the XRF technique allows for quick initial verification of the quality of the asphalt modified with various types of additives.

The chemical composition of the studied materials determined with ED-XRF is presented in Table 2.

The chemical composition of asphalts was dominated by the hydrocarbons and sulfur, reaching 99.9%. Pollutants in the form of metals: Mo, Cu and Sn were not detected, whereas the Zn contents were marginal (5 ppm) and found only in the PMB 25/55-60 asphalt. The samples of the asphalt with WEO revealed the presence of the elements originating from the additive. The amount of detected heavy metals—tin and zinc—increased along with the concentration of WEO. They are trace amounts ranging from 30 to 60 ppm Sn and from 50 to 60 ppm Zn. However, heavy metals such as molybdenum or copper, characteristic of recycled engine oil bottom, were not detected in the tested samples. The amounts of heavy metals are marginal and should not have a negative impact on the asphalt mix properties. However the use of waste engine oil has the potential to carry out heavy metals to the environment, therefore leaching tests should be carried out. 

### 4.5. Statistical Data Analysis 

In order to determine the effect of the additive type (zeolite and WEO) on the presented binder properties, the test results were statistically analyzed using two-way ANOVA tests. The obtained results are presented in Table 3 for asphalt 20/30 and Table 4 for PMB 25/55-60. If the *p* value does not exceed the significance level *α* = 0.05 assumed in the analysis, then the given factor impact on the level change of the examined feature should be considered as statistically significant. 

The statistical analysis carried out confirmed that the change in the properties of bituminous binders was dependent mainly on the amount of dispensed waste engine oil. The value of the *p* factor for the percentage WEO amount was from 0.000 to 0.0002. 

Additionally, the percentage of zeolite additive had a statistically significant impact on the penetration and viscosity of the tested bitumens. The impact of the added clinoptilolite zeolite amount was statistically irrelevant in the case of the PMB 25/55-60 asphalt (*p* = 0.0639 ˃ 0.05) penetration and viscosity of the 20/30 asphalt (*p* = 0.0652 ˃ 0.05). The softening point was not affected by the amount of added zeolite material. Only for the PMB 25/55-60 asphalt, the impact of dispensed clinoptilolite amount turned out to be statistically significant (*p* = 0.0143 < 0.05).

The results of the ANOVA statistical analysis confirmed the analysis of the results carried out in Section 4.1, Section 4.2 and Section 4.3.

## 5. Conclusions

The research presented in the paper concerned the preliminary evaluation of possible use of waste engine oil in the zeolite foamed asphalt technology. Two types of asphalt were used in laboratory tests: 20/30 penetration grade and PMB 25/55-60 bitumen modified with polymers as well as two types of zeolites: Natural zeolite clinoptilolite and Na-P1-structure type synthetic zeolite produced from fly ashes. 

The analysis of the test results shows that the change of bitumen properties is affected to a greater degree by the addition of WEO than by the addition of water-soaked zeolites, which was confirmed by the results of the statistical analysis ANOVA.

Zeolites are only a “carrier of water” and the main purpose of their use is to create the asphalt foaming effect, which in consequence allows to reduce the production and compaction temperatures of the asphalt mix. 

The asphalt binders with the WEO addition had lower dynamic viscosity and softening point but higher penetration. The highest decrease in the viscosity and softening point was recorded in the samples with 3% WEO addition. Increasing the concentration to 5% and 7% WEO caused a further change in the tested parameters with lower dynamics of changes. In turn, the growth of penetration that occurred while increasing the amount of WEO addition was proportional. Research results indicates the potential possibility of using waste materials such as waste engine oil in the asphalt mix produced in the zeolite-foamed asphalt technology with RAP.

In order to verify the preliminary results of the tests, further research including the assessment of the physical and mechanical properties of asphalt mix produced in the zeolite-foamed asphalt technology with RAP and WEO addition is required. 

## Figures and Tables

**Table 1:** Properties of the basic bitumen.

Test	Specification	Result	
		20/30 Asphalt	25/55-60 Asphalt
Penetration (25 °C; 0.1 mm)	EN 1426:2009	21.1	29.3
Viscosity at (135 °C), mPa·s	ASTM D 4402	377.0	593.5
Softening point, °C	EN 1427:2009	63.8	68.0

**Table 2:** Chemical composition of the studied materials.

		WEO 5W40	35/50 Asphalt (A)	PMB 25/55-60 (B)	A + 3% WEO	A + 5% WEO	A + 7% WEO	B + 3% WEO	B + 5% WEO	B + 7% WEO
Al	%	0.038	0.027	0.025	0.025	0.027	0.024	0.026	0.024	0.024
Si	%	0.066	0.007	0.007	0.005	0.006	0.007	0.007	0.004	0.006
P	%	0.089	0.035	0.035	0.038	0.041	0.039	0.038	0.039	0.039
S	%	0.186	3.600	3.461	3.465	3.370	3.379	3.341	3.304	3.267
**Ca**	**%**	**0.208**	**0.013**	**0.014**	**0.019**	**0.025**	**0.026**	**0.020**	**0.023**	**0.025**
V	%	nd *	0.026	0.024	0.025	0.025	0.024	0.024	0.023	0.023
Ti	ppm	4.1	nd	nd	nd	nd	nd	nd	nd	nd
Fe	%	0.002	0.006	0.005	0.006	0.005	0.005	0.005	0.005	0.005
Ni	%	nb	0.006	0.006	0.006	0.006	0.006	0.006	0.006	0.005
**Zn**	**ppm**	**680**	**nd**	**5**	**30**	**40**	**50**	**30**	**40**	**60**
**Sn**	**ppm**	**60**	**nd**	**nd**	**50**	**60**	**60**	**60**	**60**	**60**
CH_2_	%	99.338	96.280	96.422	96.403	96.485	96.477	96.525	96.562	96.595

* nd: not deleted.

**Table 3:** ANOVA analysis on the parameters of the waste engine oil (WEO)-modified asphalt.

Asphalt 20/30					
Na-P1 Zeolite					
Penetration test					
Source	*SS*	*df*	*MS*	*F*	*p*
Percentage WEO addition	1808.72	2	904.36	696.02	0.0000
Percentage zeolite addition	65.00	3	21.67	16.67	0.0026
Error	7.80	6	1.30	-	-
Total	1881.51	11	-	-	-
Softening point test					
Source	*SS*	*df*	*MS*	*F*	*p*
Percentage WEO addition	20.91	2	10.45333333	73.5	0.0001
Percentage zeolite addition	0.99	3	0.328888889	2.3125	0.1758
Error	0.85	6	0.142222222	-	-
Total	22.75	11	-	-	-
Dynamic viscosity test					
Source	*SS*	*df*	*MS*	*F*	*p*
Percentage WEO addition	11257.64	2	5628.82	189.88	0.0000
Percentage zeolite addition	1217.31	3	405.77	13.69	0.0043
Error	177.86	6	29.64	-	-
Total	12652.81	11	-	-	-
Clinoptilolite zeolite					
Penetration test					
Source	*SS*	*df*	*MS*	*F*	*p*
Percentage WEO addition	1636.14	2	818.07	751.97	0.0000
Percentage zeolite addition	43.76	3	14.59	13.41	0.0045
Error	6.53	6	1.09	-	-
Total	1686.43	11	-	-	-
Softening point test					
Source	*SS*	*df*	*MS*	*F*	*p*
Percentage WEO addition	19.46	2	9.73	143.56	0.0000
Percentage zeolite addition	0.86	3	0.29	4.25	0.0626
Error	0.41	6	0.07		
Total	20.73	11			
Dynamic viscosity test					
Source	*SS*	*df*	*MS*	*F*	*p*
Percentage WEO addition	12075.28	2	6037.64	132.13	0.0000
Percentage zeolite addition	569.54	3	189.85	4.15	0.0652
Error	274.18	6	45.70	-	-
Total	12919.00	11	-	-	-

*SS*: Sum of the squared deviations; *df:* Degree of freedom; *MS:* Mean square; *F*: F-value; *p*: P-value.

**Table 4:** ANOVA analysis on the parameters of the WEO-modified asphalt.

Asphalt PMB 25/55-60					
Na-P1 Zeolite					
Penetration test					
Source	*SS*	*df*	*MS*	*F*	*p*
Percentage WEO addition	1419.98	2	709.99	1811.56	0.0000
Percentage zeolite addition	16.06	3	5.35	13.66	0.0043
Error	2.35	6	0.39	-	-
Total	1438.40	11	-	-	-
Softening point test					
Source	*SS*	*df*	*MS*	*F*	*p*
Percentage WEO addition	40.13	2	20.06	91.66	0.0000
Percentage zeolite addition	2.52	3	0.84	3.83	0.0759
Error	1.31	6	0.22	-	-
Total	43.96	11	-	-	-
Dynamic viscosity test					
Source	*SS*	*df*	*MS*	*F*	*p*
Percentage WEO addition	49484.32	2	24742.16	75.72	0.0001
Percentage zeolite addition	9364.63	3	3121.54	9.55	0.0106
Error	1960.54	6	326.76	-	-
Total	60809.49	11	-	-	-
Clinoptilolite zeolite					
Penetration test					
Source	*SS*	*df*	*MS*	*F*	*p*
Percentage WEO addition	1303.20	2	651.60	604.81	0.0000
Percentage zeolite addition	13.57	3	4.52	4.20	0.0639
Error	6.46	6	1.08	-	-
Total	1323.24	11	-	-	-
Softening point test					
Source	*SS*	*df*	*MS*	*F*	*p*
Percentage WEO addition	23.83	2	11.91	44.92	0.0002
Percentage zeolite addition	6.71	3	2.24	8.43	0.0143
Error	1.59	6	0.27	-	-
Total	32.12	11	-	-	-
Dynamic viscosity test					
Source	*SS*	*df*	*MS*	*F*	*p*
Percentage WEO addition	42428.67	2	21214.33	371.10	0.0000
Percentage zeolite addition	3402.75	3	1134.25	19.84	0.0016
Error	343.00	6	57.17	-	-
Total	46174.42	11	-	-	-

*SS*: Sum of the squared deviations; *df:* Degree of freedom; *MS:* Mean square; *F*: F-value; *p*: P-value.
